# Alternative Traits for Genetic Evaluation of Mastitis Based on Lifetime Merit

**DOI:** 10.3390/genes15010092

**Published:** 2024-01-12

**Authors:** Gabriel Leitner, Shlomo E. Blum, Oleg Krifucks, Yaniv Lavon, Shamay Jacoby, Eyal Seroussi

**Affiliations:** 1 National Mastitis Reference Center, Department of Bacteriology, Kimron Veterinary Institute, Bet Dagan 50250, Israel; leitnerg1@gmail.com (G.L.); shlomobl@moag.gov.il (S.E.B.);; 2 Israel Cattle Breeders Association, Caesarea 3781500, Israel; yaniv@icba.co.il; 3 Institute of Animal Science, Agricultural Research Organization (ARO), HaMaccabim Road, Rishon LeTsiyon 7528809, Israel

**Keywords:** dairy cow, milk, genetic analysis, Israeli Holsteins, mastitis, immunogenetics

## Abstract

Genetic selection has achieved little progress in reducing mastitis incidence. Mastitis traits are problematic due to the lack of sensitivity of the data and reliance on clinical diagnosis, often missing subclinical cases, and/or on monthly somatic cell count (SCC) measurements. The current measure for mastitis is the lactation average of the somatic cells score (LSCS). We studied two datasets: (1) 148 heifers divided into non-intramammary infected, sub-clinically infected and clinical mastitis groups; (2) data from 89,601 heifers from Israeli Holsteins through the same period divided into “udder healthy” (UH) and “non-healthy” (UNH) by a threshold of SCC 120,000 cells/mL in all nine monthly milk recordings. In study 1, non-infected heifers had significantly (*p* < 0.05) more partum, production days and overall lifetime milk production compared to clinical and sub-clinically infected. In study 2, UH heifers (20.3%) had significantly higher (*p* < 0.01) lifetime milk, production days, and lactations. Subdividing datasets by sires, the same analyses detected differences in percentages of UH daughters between the sire groups. Lifetime milk production correlated (r = +0.83, *p* < 0.001) with udder health status. SCC threshold of less than 120,000 cells/mL during all first lactation measurements indicated healthy udder, providing a valuable insight that this dichotomous trait is advantageous for calculating lifetime net-merit index (NM$) over LSCS.

## 1. Introduction

Mastitis, the inflammation of the mammary gland, is one of the most significant causes of economic loss in the dairy industry worldwide [1]. Economic losses due to mastitis were estimated to be approximately USD 2 billion in the USA [2,3] and EUR 3 billion in the EU [4]. Losses are due to drugs, labor, discarded milk, reduced price paid for low quality milk with high somatic cell count (SCC), and above all, increased culling. Culling, or removal of cows from the herd and their replacement by first-calf heifers, is an integral part of a dairy herd management plan. With low genetic value and/or physiologically lower producing cows, voluntary culling of older cows is needed to support a producing and economically balanced herd. On the other hand, involuntary (early) culling of profitable cows, due to disease, infertility, mastitis, and high SCC, increases the need for replacement heifers, and is often at the expense of voluntary culling, thus leading to less profitable or genetically valuable cows that remain longer in the herd. The replacement rate (the percentage of voluntary, planned replacement of cows) in intensive dairy herds depends on the potential of cows to remain profitable over time and the cost of a replacement heifer, which is estimated to be approximately USD 1500–2000 in the US [5,6,7]. This encompasses expenses associated with raising a calf for a period of 23–25 months until its first parturition, a reduced milk yield of approximately 15% during the first lactation compared to subsequent ones, and the duration required for a replacement heifer to recoup the investment made. Currently, the replacement rate stands at approximately 33% in the US [8] and 29% in Canada [9], meaning an average production lifecycle of only 2.2–2.5 lactations/cow. Both management and genetic factors influence voluntary and involuntary culling; however, this is largely a “hidden cost” in dairy production that is hard to keep track of and to estimate. The reason for culling a cow is often multifactorial, including parity, pregnancy, stage of lactation and herd management practices. The specific reason for culling may remain unreported, or multiple reasons may be reported for the same cow, such as low milk yield, infertility, clinical or chronically subclinical mastitis. Pinedo et al. [10] compared the dynamics of reason-specific culling risk between different genetic groups, reporting that out of 30–33% replacement, in 50% of the cases no reason for culling was reported, or cows died with no registered cause of death. Nevertheless, mortality has been suggested to be associated with herd levels of respiratory disease, lameness, antibiotic use for treating sick cows and calving [11], whereas the two major causes of culling were reproductive disorders and mastitis [12].

The leading causes of mastitis are intra-mammary bacterial infections. Penetration of bacteria into the mammary gland occurs mostly through the teat canal and is a crucial step in the development of mastitis. The most common mastitis pathogens are also part of the normal cows’ skin microbiota, including the external part of the streak canal, and are also present in the cows’ environment. Regardless of environmental conditions and management and milking practices, most cows/glands in a herd are usually not infected at any time point. Multiple physiological, anatomical, and innate immune factors prevent intra-mammary infections—genetics must therefore play a key role. Even in the event of bacterial penetration through the teat canal, inflammation is not always triggered, owing to early bacterial elimination by the mammary gland’s innate immune system, such as killing by leucocytes and phagocytosis. Because it is practically impossible to measure the rate of success of the udder immune system in early elimination of bacteria (i.e., rate of mastitis prevention), increased SCC is used as a surrogate, measuring in fact the “lack of success”.

Studies evaluating the influence of genetic traits on mastitis incidence are hindered by the difficulties inherent in diagnosis and lack of proper reporting of cases. This is even more pronounced concerning subclinical mastitis, in which no obvious clinical signs can be seen. In these cases, mastitis is detected only by increase in milk SC. Research studies usually use cow or quarter-level markers of inflammation (e.g., SCC or the surrogate Californian mastitis test [CMT]), combined or not to bacteriological exams. But in real commercial dairy farms, data on subclinical mastitis prevalence is often scarce. Monitoring, when performed, relies on monthly (or less frequent) SCC evaluation of cows as the main or single indicator for subclinical mastitis, which lacks sensitivity for the following reasons. First, in most cases only one gland quarter is affected. Mild increase of SCC in only one of the four quarters can be easily missed when evaluating SCC at the cow level only, especially when the common 200,000 cell/mL threshold is considered. Second, in some clinical cases, especially when the cow is treated early, the SCC may decrease within days from infection to less than the above threshold, and if the infection occurs between two routine monthly milk recordings it will be missed unless manually recorded. And last, in many cases, a bacteriology test is not performed; therefore, the data available for analysis are the severity and time of inflammation (i.e., increase in SCC), which is a result of the bacteria type, the host immunology response, and their interaction.

The purpose of dairy sire pedigree records is to keep track of bulls’ genetic value and increase the success of genetic selection in dairy herds. This is done by evaluating measured differences between sires’ progenies and is based on large datasets including several daughters spread over various farms. Genetic selection is based on indexes, combinations of traits with genetic potential and economic importance. Conducted in many countries, sire pedigree-based selection programs lead to herd-wide improvements such as increased milk yields, fat and protein contents, reduced birth mortalities, etc. However, the success in reducing the incidence of mastitis has been limited. The current index for mastitis selection is the Lactation Average Somatic Cells Score (LSCS), which has a lower weight in the overall selection index. Using mean SCC and selecting for a low index value results in no reduction of mastitis occurrence [13]. Alternatives to a single mean SCC value are, for instance, the use of two consecutive SCC measurements [14], SCC at >45 days of lactation [15], and seven SCC values at distinct stages of lactation [16]. Kirsanova et al. [17] used eight SCC-based traits, and even though heritability was low, compared to LSCS, a strong genetic correlation among the traits was found suggesting that better udder health can be improved by better breeding programs.

This approach assumes that there is a uniform response of SCC to any intramammary-infecting bacteria and does not account for different intensities and patterns of inflammatory reaction that are pathogen-related, nor for the success in actually mitigating infections (bacterial cure) and does not account for incidence frequency of mastitis. Increased SCC usually reflects a mammary immune response to an intramammary infection and thus it is used as a proxy for udder health and a qualitative measurement included in indexes for breeding programs. In fact, however, actual assessment of udder health cannot be limited to simple SCC measurement because the physiological, immunological, and inflammatory response in the mammary gland is strongly pathogen and time related. Generally, Gram-negative species elicit clinical mastitis, with a strong polymorphonuclear response that is often successful in eliminating the infection within a short time. This could be regarded as a “positive” immune response, which normally ends up with bacterial clearance and return to a physiological state. In these cases, SCC level may remain considerably higher than normal for 10–50 days [18], which would then be recorded in one to two monthly milk recordings with a large effect on LSCS. On the other hand, mastitis caused by many Gram-positive bacteria tends to be subclinical and to become chronic, while the immune system fails to mitigate the infection, and SCC remains moderately high over time. This could be regarded as a “negative” response because the immune response failure in clearing the infection is associated with lower milk yield and quality, and yet, because SCC is only moderately increased and for a longer period, LSCS is affected at a lower magnitude than it would be with clinical mastitis. Even though in dairy herds, chronic mastitis cases exceed clinical ones and have a higher accumulative effect on udder health and milk production, these are overlooked when using the current LSCS-based selection methods alone. To differentiate between a “positive” and a “negative” immune response, LSCS should not stand alone as a trait and should be accompanied by time span of SCC elevation. Moreover, the actual prevention of bacterial penetration into the mammary gland is also the utmost desired characteristic, which can also influence overall incidence of mastitis and the level of udder health. This comprises anatomical, physiological, and immunological cow traits.

The objective of this study was to explore the use of an SCC threshold of <120,000 cells/mL in each single monthly milk recording throughout the first lactation as a trait that can be used for sire selection to improve udder health. We have previously shown that below this threshold the probability of intramammary infection is very low, and it thus divides between “udder healthy” (UH) and “non-healthy” (UNH) cows [19]. This study was conducted on two levels. One focused on a single herd and included a longitudinal monitoring of heifers through 2015–2022 from first lactation up to culling. The second included the analysis of all Israeli heifers registered in the Israeli herd book during the same period. An analysis of siring bulls was performed to investigate genetic effects.

## 2. Materials and Methods

### 2.1. Experiment 1: Single Herd Follow Up

The study was conducted on one herd of 200 Israeli Holstein dairy cows producing an average milk yield of >11,500 L/305 lactation days. The dairy parlor was equipped with a computerized AfiFarm Herd Management system and AfiLab milk analyzer (Afimilk, Afikim, Israel), providing on-line data on gross milk composition and conductivity. Cows were milked thrice daily at 4:00, 13:00 and 20:00. Routine monthly milk yield, milk composition and SCC were recorded by the Israeli Cattle Breeders Association (ICBA, Caesarea, Israel). Gross milk composition analysis including fat, protein and lactose content was performed with the Milkoscan FT+ (Foss Electric, Hilleröd, Denmark). SCC counts were performed with Fossomatic FC (Foss Electric, Hilleröd, Denmark). Seventy to eighty percent of the female calves are usually raised on Israeli farms as replacement for older cows. Female calves were raised in similar age groups, artificially inseminated at 13–15 months and transferred to a parturition yard 3 weeks before the expected calving. Post calving, heifers were moved to the first-calve heifers’ group through the first lactation. Through 2015–2017, all heifers from calving were included in the follow-up study. Any changes in udder health were recorded and tested by the researchers using the California mastitis test (CMT) of individual mammary quarters, and samples for bacteriologic culture were taken when CMT score was >1. The same procedure was conducted following the routine monthly tests for each of the heifers with SCC > 80,000 cells/mL. When these tests indicated an intramammary infection with inflammation or inflammation alone, two additional samples were taken 2–3 days apart. At 70–120 days in milk (DIM)—peak of lactation—heifers were tested for bacterial infection and milk composition, including SCC, at the mammary quarter level. After verification of each quarter’s condition, composed milk of all uninflamed and uninfected glands (no bacteria found in culture and SCC < 50,000 cells/mL) was sampled during the afternoon milking, and milk composition, including SCC and cell distribution, were tested by flow cytometry (FACs Calibur, Becton-Dickinson, San Jose, CA, USA) as described by [18]. Milk bacteriologic cultures were performed according to the International Dairy Federation [20]. Briefly, ten microliters of each milk sample were inoculated onto blood agar (enriched with 5% of washed sheep red blood cells) and MacConkey agar plates (Bacto-Agar, Difco Laboratory, Becton Dickinson, Le Pont de Claix, France). The plates were incubated at 37 °C and examined for bacterial growth after 18 and 42 h. General data on daily and lactation milk yield, milk composition, number of artificial inseminations to pregnancy, length of lactation, medical events and culling during first lactation were recorded from the on-line and ICBA Herd Book. Clinical mastitis was treated with antibiotics. From the second lactation and until removal of the cow from the herd, up to 9 lactations, the number of productive days, lifetime milk production and number of calvings were recorded from the on-line and ICBA Herd Book. Further details regarding this heifer dataset are given in Supplementary Materials (File S1), and have been previously described [21].

### 2.2. Experiment 2: Analysis of Israeli Herd Book Data

This experiment was performed using a dataset extracted from the Israeli herd book (ICBA). The dataset included records of all heifers at first lactation in Israeli dairy herds through 2015–2017 and included 89,601 heifers, which were classified into one of the following subsets according to SCC during the first lactation: one subset included heifers with SCC < 120,000 at each one of the nine monthly milk recordings, which were considered UH, whereas heifers with SCC above that threshold in any one of the milk recordings were grouped in the second subset, considered UNH. First, the analysis compared the two subsets’ total productive days, lifetime milk production and number of calvings. Second, the sire (cow father) element was added to the model, and the same parameters were compared for each sire with >100 heifers in the dataset.

### 2.3. Statistical Analysis

Three analyses were performed. Overall performance of uninfected, clinical, and subclinical infected heifers at first lactation: this analysis was conducted once for specific dairy farm and once for total primiparous cows that were milking during the years 2015–2017 and registered in the Israeli herd book.

Multivariable models were designed with a logistic model statement using the GLIMIX procedure of SAS, with productive days, lifetime production of milk and number of calvings as the dependent variable, as previously described [19]. The model tested separately the number of productive days, lifetime production of milk and number of calving in cows with low SCC during first lactation (SCC < 120,000) and in cows with higher SCC levels. The analysis was performed with the general form: productive days, lifetime production of milk or number of calving (Equation (1), where g120 is number of cows with SCC levels higher or lower than 120,000):

number of calving = intercept + g120 + error(1)

At the time of analysis of different parameter values, all the herds’ cows that were still in the herd were included. Differences between sires were tested in 79 sires with more than 100 heifers in all the data. Differences between sires are presented as means for lifetime production between cows with SCC levels higher or lower than 120,000. To compare levels within a variable, we ran the Bonferroni adjustment for multiple comparisons.

## 3. Results

### 3.1. Experiment 1: Single Herd Follow Up

A hundred and forty-eight first heifers were included in the follow up. The overall performance (model 1) of non-infected, clinical, or sub-clinical infected heifers at first lactation is summarized in Table 1. Time and season of partum did not influence the incidence of udder infection. Sixty-five heifers (43.9%) did not have mastitis at all: SCC was <80 × 10^3^ cells/mL in all monthly milk recordings, and therefore, with a mean lactation SCC of 62 ± 6 × 10^3^ cells/mL. Eighty-three heifers (56.1% of heifers in the study) had either sub-clinical (*n* = 64, 43.2% of heifers in the study) or clinical (*n* = 19, 12.8% of heifers in the study) mastitis in one or more glands. Among heifers with a clinical infection, five also had a sub-clinical infection in another quarter. Different coagulase-negative staphylococci were the main bacteria causing sub-clinical intramammary infections (IMI), while *Streptococcus* spp. were the main bacteria causing clinical ones (Table 2). Among infected heifers, 66.3% were either already infected before or became infected within days from parturition, and 33.7% became infected during the lactation. None of the sub-clinical infections developed into a clinical infection, and all new infections were in different glands in heifers. Chronic infections developed until the end of lactation, i.e., no “spontaneous cure” was observed in this set of animals. The mean SCC in the first lactation of either sub-clinically or clinically infected heifers was significantly higher than that of non-infected ones (*p* < 0.001, Table 1). Culling of animals was an outcome of intra-mammary infections in 10.9% (*n* = 7) of sub-clinically and in 57.9% (*n* = 11) of clinically infected heifers. Nine heifers were culled at the end of the first lactation due to infertility (*n* = 6) or due to excessively large udders (*n* = 3). Overall, 25 heifers (17%) were culled during the first lactation; of these, 72% were culled due to mastitis, 20% due to infertility and 8% due to other reasons.

When compared between non-infected vs. infected cows, those without an intra-mammary infection during the first lactation had significantly more parturitions, production days, and overall produced more energy-corrected milk (ECM) (Table 3). When the type of intra-mammary infection was considered, these production parameters were more significantly affected among clinically infected cows (Table 4). Moreover, even though all clinically infected cows were treated with antibiotics, approximately 70% (13/19) of these animals were culled before second partum. Although some were treated with antibiotics, sub-clinically infected heifers had less parturition and produced fewer ECM kg of milk than non-infected animals.

### 3.2. Experiment 2: Analysis of Israeli Herd Book Data

The dataset included a total of 89,601 first lactation heifers’ entries, with 79 sires with >100 heifers identified. At the time of analysis, above 80% of heifers had been culled. Therefore, lifetime milk production and number of lactations for the remaining 20% of the heifers that were still in the herd were a snapshot. Overall, 18,165 heifers (20.3%) within the dataset were classified as UH based on the SCC threshold described in the Materials and Methods section—all the others (*n* = 71,436, 79.7%) being classified as UNH. Lifetime milk production, productivity days, and number of lactations were significantly higher (*p* < 0.001) in UH compared to UNH heifers (Table 5). On average, UH heifers produced 1736 kg milk and 0.27 calves and had 13 on-farm production days more than UNH ones.

**Figure 1:**
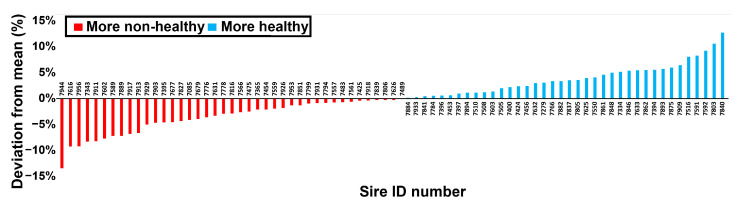
Deviation of the percent of udder healthy (UH) from total daughters (mean 19.23%) for each of the 79 sires. Statistical data and standard deviation are further described in Table S1.

All 79 sires had both UH and UNH daughters. Varying in a magnitude of +/−13%, on average there were 19.2% of UH out of all the daughters among the different sires. The distribution of the deviation from this average is shown in Figure 1.

**Figure 2:**
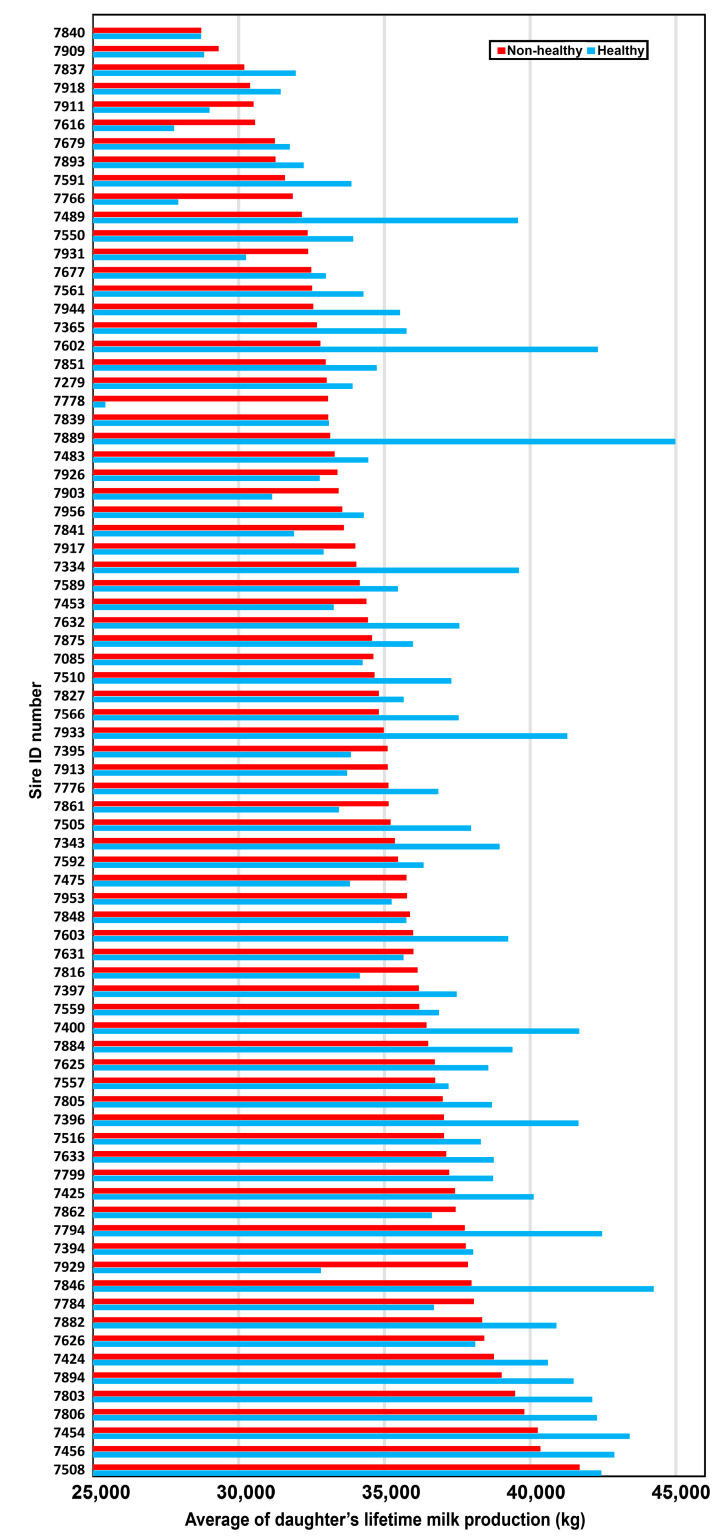
Comparison between the average lifetime milk production of healthy (UH) and non-healthy (UNH) daughters calculated for each of the 79 sires. Sires were sorted by their UNH values. Each average is represented by bars colored blue (UH) or red (UNH). Statistical data and standard deviation are further described in Table S1.

**Figure 3:**
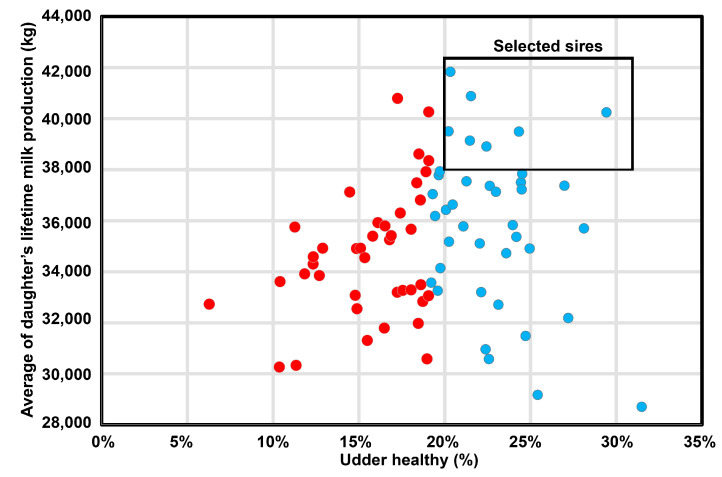
The percentage of udder healthy (UH) vs. lifetime milk production calculated for each of the 79 sires. Superior sires with the UH > 20% and the lifetime milk production >38,000 kg are denoted by a box. Each bull is represented by a dot colored blue (UH) or red (UNH) as in Figure 1.

Lifetime milk production of udder healthy and non-healthy daughters was calculated separately for each sire (Figure 2), showing large variability among sires. Overall, average lifetime milk production was 35,603 (37,632 and 35,896 for udder healthy and non-healthy, respectively). A trend was found for the number of lactations between UH and UNH daughters, with mean number of lactations 3.01 (3.24 and 2.97, respectively) (Table 5). A low correlation (r = 0.125) was found between lifetime milk production and % UH. However, examination of percentage of udder healthy and lifetime milk production of each of the 79 sires indicated several superior sires (Figure 3). There was a positive correlation of r = 0.83 (*p* < 0.001) between the number of lactations and lifetime milk production.

## 4. Discussion

Research of life-time merit of dairy cow is limited because a long period of follow-up throughout the life of the cow is required. Yet, this trait is of utmost importance for farmers [22,23,24]. Genetic improvement of livestock by offspring selection is as old as animal domestication for agriculture. On a large scale, selection uses indexes, weighting different phenotypes, such as productivity, product quality, external traits, endurance, reproduction success, etc., depending on cultural, geographical, and economic needs and practices. Selection in dairy cows has focused over the years on traits such as milk yield, fat and protein contents, reproduction success rates, and endurance. Traits are selected based on overall economic importance to the dairy sector and the Predicted Transmitting Ability (PTA). Because a dairy cow requires a significant initial investment before becoming productive and profitable, its genetic value should be evaluated for the long-term, with lifetime productivity and endurance as the main goals. In the U.S., for instance, the national selection index, lifetime net merit $ (NM$), is a measure of expected lifetime profit of dairy cows as compared to breed base cows with traits that are genetically and economically important [25].

The PTA for mastitis prevention is generally regarded low. One likely reason is because the evaluation of this trait does not account for actual udder infection or for the type of infecting bacteria, but rather on the inflammation outcome as evaluated by SCC alone. Mastitis is a response to an intra-mammary infection, which follows bacterial invasion into the mammary gland via the teat canal. Selection should therefore target highly resistant cows, with the traits better preventing pathogen invasion and/or faster elimination before an infection actually develops.

We argue that mean SCC is not a reliable indicator of mastitis resistance for sire selection, because it does not account for the variability of SCC responses to different mastitis pathogens. This assumption is problematic for several reasons. First, the 50,000–100,000 cells/mL SCC threshold may not accurately separate infected from non-infected cows, as some non-infected cows may have elevated SCC due to factors other than mastitis. Second, as reflected by SCC, the intensity and duration of the inflammatory response depend largely on the type of pathogen involved. Mostly gram-negative pathogens cause a rapid and substantial increase in SCC, usually for short duration, whereas others, such as CoNS, cause a mild but chronic increase in SCC. Third, some cows may receive treatment or have their infected gland dried off, which would lower their SCC. Fourth, and perhaps most importantly, SCC is a beneficial natural mechanism for eliminating intra-mammary bacteria, so a high SCC for a short period may be preferable to a moderate SCC for a long period (chronic infection). Therefore, any approach that uses average SCC without accounting for the dynamic and diverse nature of the inflammation process to intra-mammary infections is likely to fail in selecting mastitis-resistant cows.

The Israeli breeding program is monitored by the Israeli Breeding and Herd Book Committee that frequently updates the Israeli Selection Index, which includes coefficients for milk, fat, protein and other traits that are computed to maximize expected farmer profit [26]. Current index (PD20) coefficient for somatic cell score (SCS) is computed so that expected changes for SCS would be close to zero (Equation (2)):

PD20 = 9.94 (Fat kg) + 19.88 (Protein kg) + 2 (Protein/fat ratio) − 300 (SCS) + 26 (Female fertility %) + 0.6 (Days of herd life after first calving) + 10 (Persistency %) − 3 (Dystocia %) − 6 (Calf Mortality %).(2)

Thus, for example, the positive index value of an international sire with best SCS score (−0.77, #5940, Cannonball, HO840003133157816 [27]) is substantially increased (32%) by the SCS trait. For the 79 bulls tested in this study, the rate of UH daughters was highly and negatively correlated (r = −0.74) with SCS. Thus, the current index does deliver most of the selection potential encoded in an index coefficient that would be based on the UH trait. Yet, with high incidence of cows that immunologically collapse during the first lactation, it may be that selection by this trait would better avoid such incidences.

In this study, we suggest using a dichotomous value (healthy vs. unhealthy) instead of the mean LSCS to classify cows based on their mammary gland health. We set the threshold of first lactation monthly SCC at <120,000 mL for each one of the measurements, which indicates a low probability of infection or inflammation in the udder. We consider the end point values to be important for a better evaluation of longevity and life throughput, as well as the variability among sires. Archer et al. [28] showed that high SCC early in the first lactation reduces longevity and increases culling risk, implying that cows need to survive beyond the first lactation to cover their rearing costs. Our results suggest that using a dichotomous first lactation monthly SCC of <120,000 mL in NM$ index instead of LSCS is advantageous. In most of the 79 sires tested, healthy cows had higher lifetime milk production and lactation number than non-healthy heifers in first lactation, but with different magnitudes. This indicates that heifers that maintain healthy udders throughout the whole first lactation are more likely to stay in the farm and have higher NM$. Our results also suggest that there is large variation among the 79 sires tested in ECM, implying that sire selection can be improved by considering long-term and lifetime production.

Lack of correlation between lifetime milk production and % “udder healthy” is expected because of different genes involved in milk production, whereas mastitis events are negatively influenced by the individual genetics’ potential. However, by the examination of percentage of “udder healthy” and lifetime milk production of each sire, several superior sires were found. This suggests that decreased replacement, and increased lifetime and lactations can be achieved by selection for sires with a high number of “udder-healthy” heifers according to an SCC threshold of <120,000 cells/mL in each single monthly milk recording throughout the first lactation.

## 5. Conclusions

This study presents insights on life-time merit follow-up, concluding that the immunological genetics of the cow have a major effect on lifetime merit. Thus, current selection for protein and fat milk content might promote incidence of cows that at first present superior content but later collapse, without even covering their costs. Therefore, for better selection, we suggest taking udder health into account using a dichotomous trait that is based on an SCC threshold of less than 120,000 cells/mL during all first lactation measurements, which indicates a low probability of infection or inflammation in the udder.

## Figures and Tables

**Table 1:** Average overall performance (model 1) of non-infected, clinical, or sub-clinical infected heifers at first lactation.

Parameter	Intra-Mammary Infection Type			*p* [F]
	Non-Infected (*n* = 65, 43.9%)	Subclinical (*n* = 64, 43.2%)	Clinical (*n* = 19, 12.8%)	
Culled due to mastitis (%)	-	7 (10.9%)	11 (57.9%)	*p* < 0.001
Culled due another reason	3 (4.7%)	6 (11%)	-	NS ^1^
Finished first lactation	62 (95.3%)	50 (78.1%)	8 (42.1%)	*p* < 0.001 ^2^
Mean SCC (×10^3^ cells/mL)	62 ± 6	263 ± 35	562 ± 114	*p* < 0.001

^1^ NS, *p* > 0.05. ^2^ between Non-infected to Subclinical and Clinical.

**Table 2:** Time of first detection and species of bacteria causing clinical or subclinical intra-mammary infections during first lactation.

Parameter		Intra-Mammary Infection Type		Total (%)
		Subclinical (*n* = 64, 77.1%)	Clinical (*n* = 19, 22.9%)	
Estimated time of	Pre- or immediately post-partum (0–7 DIM)	39 (69.6)	15 (55.6)	54 (65.1%)
	Lactation (30–270 DIM)	17 (30.4)	12 (44.4)	29 (34.9%)
CoNS * (number per gland)		46 (19/16/7/4)	6 (4/2/-/-)	52 (62.7%)
*Staphylococcus aureus*		1	2	3 (3.6%)
*Streptococcus* spp. (number per gland)		9 (8/1/-/-)	12 (10/2/-/-)	21 (25.3%)
*Escherichia coli*		-	7	7 (8.4%)

* CoNS, coagulase-negative staphylococci. Numbers per front-left/front-right/rear-left/rear-right mammary quarters.

**Table 3:** Mean and standard error of number of lactations, milk production days, and lifetime energy-corrected milk (ECM) of intra-mammary non-infected vs. infected heifers at first lactation.

Parameter	Intra-Mammary Infection Type		F	*p* [r]
	Non-Infected (*n* = 65)	Infected (*n* = 83)		
Number of lactations	3.65 ± 0.21	2.96 ± 0.19	7.26	0.017
Productivity days	1335 ± 80	889 ± 71	19.95	<0.001
Lifetime ECM	49,470 ± 3346	37,729 ± 2961	8.16	0.0095

**Table 4:** Mean and standard error of number of lactations, milk production days, and lifetime energy-corrected milk (ECM) of clinically vs. sub-clinically infected heifers at first lactation.

Parameter	Intra-Mammary Infection Type		F	*p* [r]
	Sub-Clinical (*n* = 64)	Clinical (*n* = 19)		
Number of lactations	3.30 ± 0.21	1.84 ± 0.39	10.58	0.003
Productivity days	1043 ± 80	383 ± 141	13.35	0.0003
Lifetime ECM	42,830 ± 3432	20,544 ± 6299	9.89	0.0043

**Table 5:** Comparison of the average and standard error of lifetime milk production, productivity days, and number of lactations of non-infected (UH) and potentially infected heifers (UNH).

Group	Cows ^1^	Number of Lactations	Productivity Days	Lifetime ECM
Udder healthy	18,165	3.24 ± 0.01	1019 ± 4.13	37,632 ± 158
Udder non-healthy	71,436	2.97± 0.005	1006 ± 2.1	35,896 ± 79
Change		0.27	13	1736
*p* [r]		<0.0001	0.008	<0.0001

^1^ Out of 89,601 entries from the Israel Herd Book.

## Data Availability

Restrictions apply to the availability of these data. The data were obtained from the database of the Israel Cattle Breeders Association (ICBA) and are available from the authors with the permission of ICBA. Nevertheless, detailed sire data are available to the public at the ICBA website https://akol.co.il/icbaapp/mivhanparim/, accessed on 9 January 2024.

## Supplementary Materials

The following are available online at https://www.mdpi.com/article/10.3390/genes15010092/s1: File S1: Dataset (includes health group; ID; number of cows; birth; partum and culling dates; number of lactations; pedigree: sire, dam, and paternal and maternal grand sires; records for the first lactation: DIM, ECM, fat and protein milk content, and mean SCC; and lifetime ECM); Table S1: Lifetime milk production of udder healthy and non-healthy daughters.
